# Exemption from Jury Duty

**Published:** 1881-09

**Authors:** 


### EXEMPTION FROM JURY DUTY.

The Georgia legislature has passed a bill exempting all practicing dentists from service on juries.—*Dental Luminary.*

The Ohio laws exempt physicians, but not dentists, although it seems to be the general practice of the officers to omit dentists in making up the lists—evidently confounding us with the "doctors." —*Ed. Reg.*

